# Supplementary material for: Single-molecule and super-resolved imaging deciphers membrane behavior of onco-immunogenic CCR5
Source: iScience. 2022 Nov 25;25(12):105675. doi: 10.1016/j.isci.2022.105675 (PMC9763858; doi:10.1016/j.isci.2022.105675)
Supplement: Document S1. Figures S1–S5 [file mmc1.pdf]

iScience, Volume *25*

Supplemental information

Single-molecule and super-resolved imaging deciphers membrane behavior of onco-immunogenic CCR5

Patrick Hunter, Alex L. Payne-Dwyer, Michael Shaw, Nathalie Signoret, and Mark C. Leake

Supplemental Information

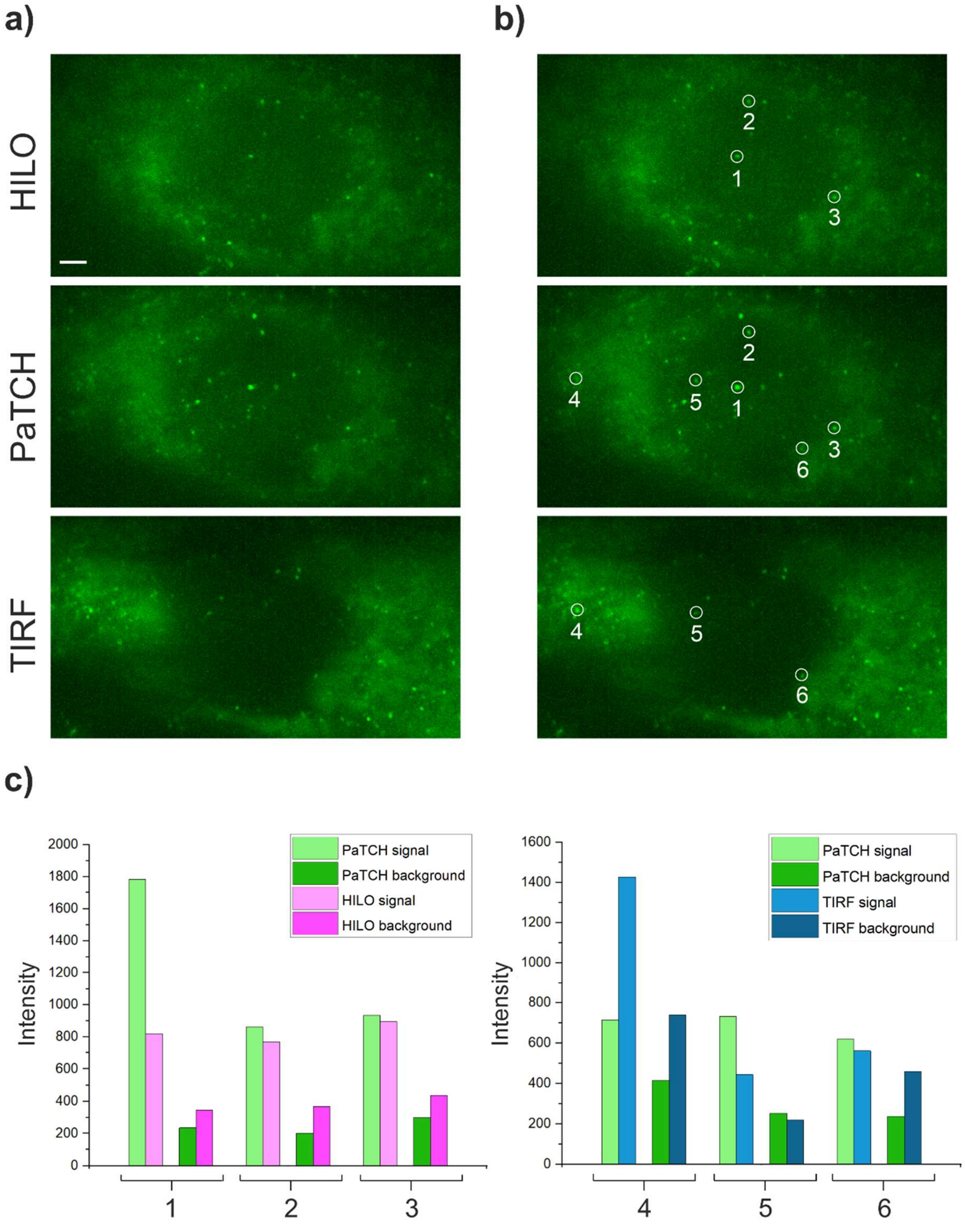

Figure S1. Comparison of HILO, PaTCH and TIRF imaging modes for the single molecule detection of GFP-CCR5 in CHO-GFP-CCR5. Related to Figure 4. *a) GFP-CCR5 expressing CHO cell imaged using HILO, PaTCH and TIRF microscopy. Thereby revealing the increased signal of puncta within the basal membrane in PaTCH when compared with HILO, whilst demonstrating the uniformity of excitation of the basal membrane in PaTCH when compared with TIRF. (Scale bar 2 µm). b) Circular overlays highlighting CCR5 assemblies present in both HILO/PaTCH images and in both TIRF/PaTCH images. Overlays are numbered to facilitate further analysis. c) Comparison of signal and background between HILO and PaTCH imaging modes, in puncta labelled 1-3, and between TIRF and PaTCH*

imaging modes, in puncta labelled 4-6. Intensity represents the raw integrated density, captured using a 6-pixel diameter circle, above a mean global background calculated using the extracellular space. Measurements of signal were taken directly over the puncta, while measurements of local background were taken adjacent to puncta. In general, puncta imaged using PaTCH benefit from a signal enhancement, relative to HILO, due to the TIRF-coupled component of illumination. Further, although TIRF is capable of providing enhanced signal relative to PaTCH, as seen in puncta 4, this restricted illumination mode results in higher background from fluorescent material close to the coverslip as well as lower signal from puncta not in close contact with the coverslip. These results combined with the result for the average signal to background ratio of 1.8 ± 0.1, 3.6 ± 0.6 and 2.4 ± 0.4 for puncta within HILO, PaTCH and TIRF respectively demonstrate the general increase in signal and reduction in background found in PaTCH when compared with HILO and TIRF.*

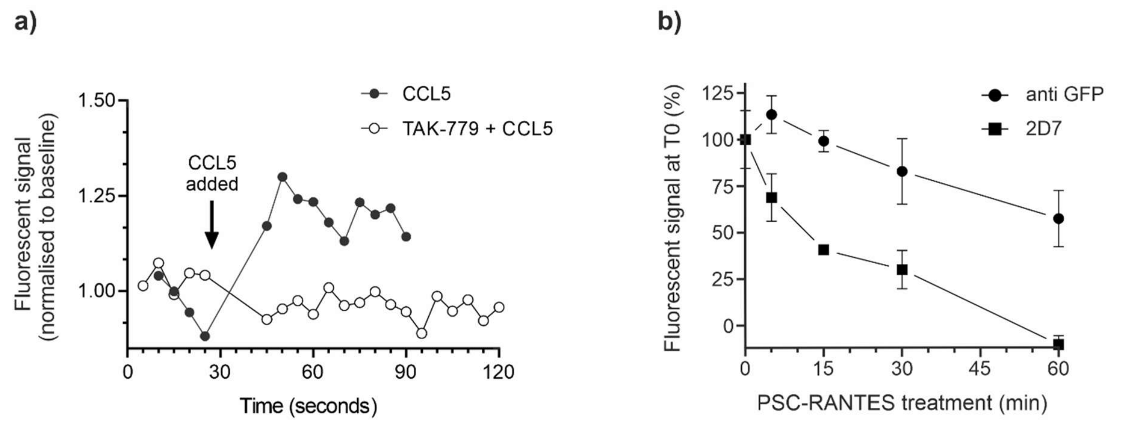

Figure S2. Increase in calcium flux, coupled with a decrease in anti GFP and 2D7 antibody binding, upon ligand stimulation confirms the functionality of GFP-CCR5. Related to Figure 3 and Figure 6. *a) Calcium flux assay in which the change in calcium associated fluorescent signal is monitored within samples of CHO-GFP-CCR5 during live exposure to 10 nM CCL5, with and without pre-exposure to the CCR5 antagonist TAK-779. Thereby revealing an increase in CCL5-associated calcium signalling in the absence of an antagonist, suggesting a functional response of GFP-CCR5 to CCL5. Values of fluorescent signal are reported normalised to the average baseline fluorescence prior to CCL5 exposure. b) Fluorescent signals associated with antibodies bound to GFP (anti GFP) and the CCR5 chemokine binding site (2D7) are measured within CHO-GFP-CCR5 cells that underwent fixation after varying levels of exposure to the super-agonist PSC-RANTES at a concentration of 100 nM. Thereby revealing a decrease in both the accessibility of the GFP epitope and the availability of the chemokine binding site, suggesting the binding of ligand and the subsequent internalisation of GFP-CCR5. Experiment was performed in triplicate and data are expressed as the mean values ± SEM.*

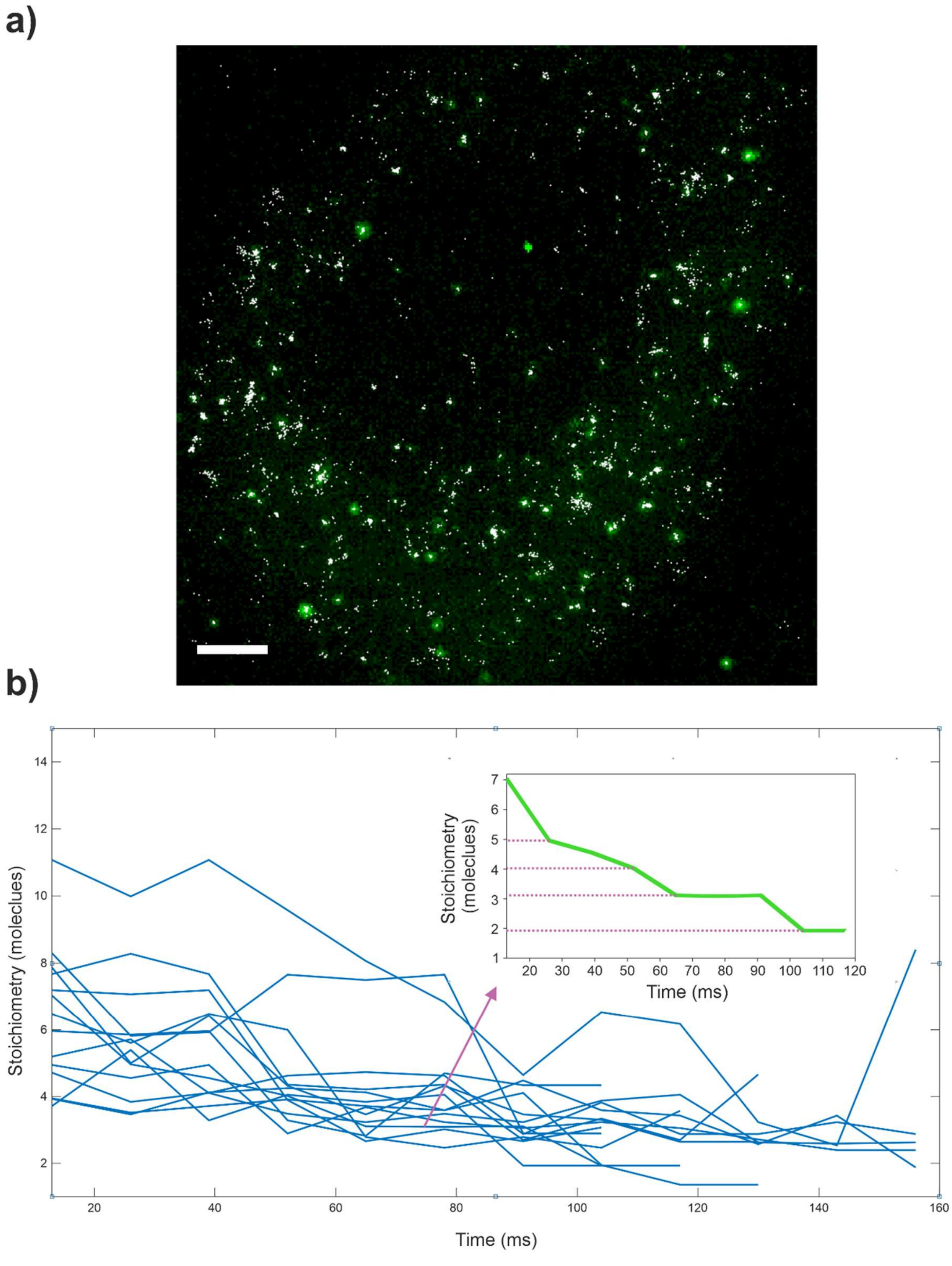

Figure S3. ADEMScode is used to detect foci within PaTCH microscopy images. Related to Figure 5. *a) GFP-CCR5 expressing CHO cell imaged using PaTCH microscopy with an overlay (white) showing tracks determined by ADEMScode tracking (MATLAB). (Scale bar 2 μm). b) Chung–Kennedy edge-preserving filtered Intensity time traces revealing the photobleaching-induced intensity decay of tracked foci towards the end of the photobleaching process. The representative traces shown here exhibit fluctuations in intensity, however this effect is accounted for in the determination of stoichiometry. Inset trace (green) shows an example of a focus whose intensity underwent decay with minimal fluctuation, dropping in a stepwise fashion from an apparent stoichiometry of 7 to 2, thereby*

supporting the accuracy of the estimated brightness of a single GFP molecule acquired from the modal brightness of monomeric GFP-CCR5.

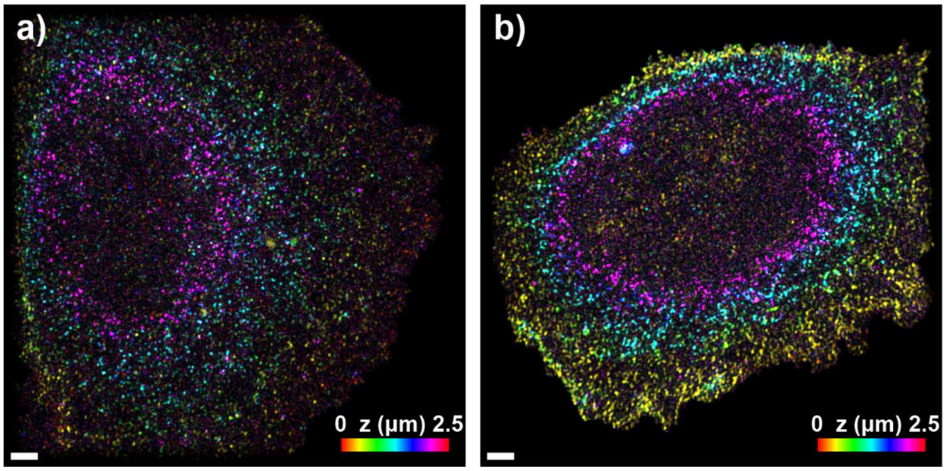

Figure S4. Comparison of cellular imaging demonstrates slight variation in brightness. Related to Figure 6. *Color depth projection of cell images shown in a) Figure 1 h) and b) Figure 6 h). Comparison of which at identical contrast settings demonstrates the slight variation in brightness that can exist between cells. This change in brightness can stem from many factors, including the natural variance of expression in this non-clonal cell model. (Scale bar 2 μm).*

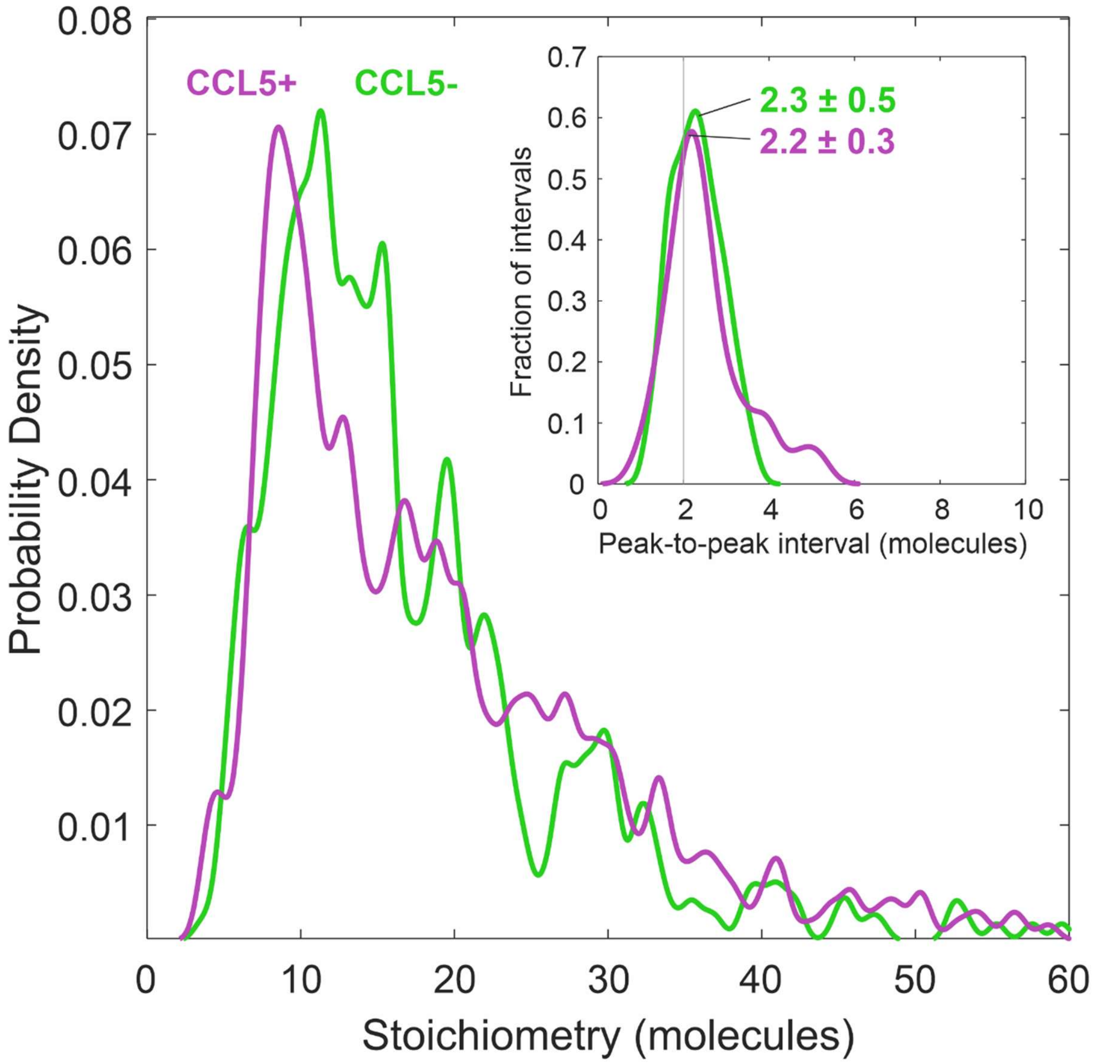

Figure S5. Periodic stoichiometry distribution indicates that CCR5 assemblies comprise dimeric subunits both before and after CCL5 exposure. Related to Figure 6. *Kernel density estimates of stoichiometry and (inset) periodic stoichiometry intervals of GFP-CCR5 associated foci before (green) and after (magenta) the addition of CCL5 (N=460 or 507 tracks respectively) detected by PaTCH microscopy in GFP-CCR5 transfected CHO cells (N=9 and 11 cells respectively). Kernel width = 0.6 molecules, corresponding to the total uncertainty in the single molecule stoichiometry, rather than statistical fluctuations. Measured intervals in probability density or stoichiometry are therefore more reliable at lower stoichiometry. (BM test for difference in periodicity, p=0.479|* NS).*
